# Brain activation patterns resulting from learning letter forms through active self-production and passive observation in young children

**DOI:** 10.3389/fpsyg.2013.00567

**Published:** 2013-09-23

**Authors:** Alyssa J. Kersey, Karin H. James

**Affiliations:** Department of Psychological and Brain Sciences, Indiana UniversityBloomington, IN, USA

**Keywords:** fMRI, brain, development, children, writing, reading, cursive

## Abstract

Although previous literature suggests that writing practice facilitates neural specialization for letters, it is unclear if this facilitation is driven by the perceptual feedback from the act of writing or the actual execution of the motor act. The present study addresses this issue by measuring the change in BOLD signal in response to hand-printed letters, unlearned cursive letters, and cursive letters that 7-year-old children learned actively, by writing, and passively, by observing an experimenter write. Brain activation was assessed using fMRI while perceiving letters—in both cursive and manuscript forms. Results showed that active training led to increased recruitment of the sensori-motor network associated with letter perception as well as the insula and claustrum, but passive observation did not. This suggests that perceptual networks for newly learned cursive letters are driven by motor execution rather than by perceptual feedback.

## Introduction

### The link between reading and writing

The ability to read and write is essential for success in today's society. As such, children begin learning these skills from a very early age. One of the first steps in learning to read is mastering letter recognition. Performance in letter recognition during this early stage has been shown to be predictive of children's literary success. For example, preschoolers' speed and accuracy when naming letters and the presence of delays in letter recognition have both been shown to predict reading skill (O'Connor and Jenkins, 1999; Lonigan et al., 2000; Stage et al., 2001). Just as letter identification has been linked to reading, writing abilities have been linked to cognitive processes including spelling (Berninger et al., 1998), text generation while composing (e.g., Berninger et al., 1991, 1992, 1994; Graham et al., 1997; Connelly et al., 2006), and the note-taking process (Peverly, 2006). Several studies have also shown interesting relationships between learning to write and improved word reading (e.g., Berninger et al., 1997, 2004, 2006; Dunn and Miller, 2009) and between writing abilities and the efficiency in learning a pseudoletter (Richards et al., 2011). Other studies have also shown that the type of motor experience can also affect letter perception. Longcamp et al. (2005b) found that older preschool children were better able to recognize letters if they had practiced writing them as opposed to typing them. These researchers proposed that the writing condition facilitated an internal model of the letter due to integration of vision, motor commands, and kinesthetic feedback, which typing does not provide. This group suggested that while typing, the children are simply building a cognitive map of the keyboard rather than gaining extensive knowledge about the letters that they learn. Similarly, adults who wrote new pseudoletters were better able to recognize the correct orientation of the character than those who typed them (Longcamp et al., 2006a). Researchers attributed this result to the representations of each pseudoletter in memory. Because the writing condition created an association between the motor system, visual system, and kinesthetic feedback, the adults were able to increase their knowledge about the spatial information for each character. The typing condition created an association between the pseudoletter and an arbitrary clue, leading to a less accurate representation of the character. Furthermore, the writing condition is a more complex motor action, suggesting that more intensive motor experiences led to enhanced visual representations.

Neuroimaging research has also suggested an important link between writing and letter perception. The visual system has been shown to process different categories of stimuli with different systems, often referred to as “functional specialization.” Whether or not this specialization is due to stimulus category or processing requirements of different stimuli is an active research question (e.g., Kanwisher and Yovel, 2009; Bukach et al., 2010), that will not be explicitly addressed here. Nonetheless, functional specialization for the visual processing of words and letters in the fusiform gyrus reveals two interrelated functional regions. The *anterior* left fusiform gyrus has been shown to be more responsive to individual letters than to letter strings, words, digits, or Chinese characters (James et al., 2005), while the *posterior* region of the left fusiform gyrus, described as the visual word form area (VWFA), responds more to letter strings and words than to individual letters (Cohen and Dehaene, 2004; James et al., 2005). Interestingly, the anterior region that underlies visual letter perception, is affected by writing experience. Adults that learned to form pseudoletters by hand, showed increased activation in this letter processing region only after pseudoletters were written, but not after typing or visual practice alone (James and Atwood, 2009). Similarly, pre-literate children (4–5 year olds) that practiced learning letters recruited this region during a subsequent fMRI scan only after printing was incorporated into their learning. Those who learned letters by visual-auditory practice (James, 2010) or by typing practice (James and Engelhardt, 2012) did not recruit this region during subsequent letter perception. These latter studies provide convincing evidence that printing letters by hand affects the neural processing of letter perception, and by extension, may have a significant effect on the development of reading skills.

Further evidence for the interaction between writing and letter perception comes from another interesting set of findings that have shown that during letter perception, motor regions of the brain are active (Longcamp et al., 2003, 2005a, 2011; James and Gauthier, 2006; James and Atwood, 2009; James and Engelhardt, 2012). Similar motor system activation has been shown during the visual perception of tools (Chao and Martin, 2000) and during verb reading (Hauk et al., 2004), demonstrating that systems that are used during action are re-activated during visual perception. It has been proposed that these networks are driven by interactions with the letters via motor experiences such as writing, leading to a similar re-activation of motor systems during visual perception (James and Atwood, 2009; James and Engelhardt, 2012).

In sum, functional specialization for letters likely develops through motor experiences during the initial stages of learning. Further, the motor experience of writing greatly enhances the response of neural substrates for letter perception more so than typing motor experience (Longcamp et al., 2005b, 2006a; James and Atwood, 2009; James and Engelhardt, 2012).

### Differences between cursive script and manuscript

Much of this previous research focused on hand-printed letters or on pseudoletters (letter-like symbols). Very little research has been performed to address the relationship between reading and writing in regards to cursive letters. Cursive letters present a unique scenario. By the time most children learn cursive writing, they have learned how to read and write the printed alphabet. Thus, learning cursive letters involves matching a new visual form to, and integrating a new motor plan into, the representation for its hand-printed counterpart. Recently, teaching cursive writing in elementary schools has met with controversy, leading in some cases, to abolishing this requirement in many American elementary school curricula. Nonetheless, little is known about the usefulness of learning cursive writing in terms of literacy outcomes, and the mechanisms that underlie this skill in children are unknown. In adults, a dissociation between processing cursive letters and hand-printed ones has been documented (Qiao et al., 2010; Longcamp et al., 2011) suggesting an important difference between the two, at least in terms of neural processing. The present study addresses the emergence of this dissociation in children first learning to write letters in cursive script.

Behavioral evidence suggests that perceiving letters that are written in cursive is more difficult than perceiving typed letters (Corcoran and Rouse, 1970; Qiao et al., 2010). Furthermore, letters written in cursive do not facilitate the speeded perception of letters that are typewritten—that is, they do not “prime” recognition (Qiao et al., 2010).

Neuropsychological studies also offer evidence for dissociations between cursive and manuscript print. Clinical observations have revealed that following a stroke, some aphasic patients display a preference for reading and writing in one modality over the other (Boone and Friedman, 1976; Williams, 1984). Williams (1984) hypothesized that because the patients who were better at reading cursive had higher combined scores on the reading comprehension task than the patients who were better at print, cursive stimuli might be more beneficial for those with less severe reading comprehension impairments, while those who are more severely impaired may benefit more from printed stimuli. Alternatively, because cursive is more difficult to perceive, the less severely damaged patients could still perform in the cursive tasks, while those who were more severely impaired could only perform when the letters were easier to perceive (manuscript print). In a case study, Hanley and Peters (1996) discuss the unique writing patterns of HN, who, following a stroke, could print uppercase letters, but struggled when it came to printing lowercase letters. However, his ability to write lowercase letters in cursive remained relatively intact. These clinical cases suggest that different mechanisms underlie, not only the perception, but also the production of cursive and printed letters.

Imaging studies have also addressed this apparent dissociation in adults. Longcamp et al. (2006b) used magnetoencephalography (MEG) to investigate the distinction between handwritten printed and cursive letters. The researchers suspected that part of the distinction in processing cursive and hand-printed letters was due to personal knowledge about motor rules involved in writing, and for this reason, they focused their study on the motor cortex. They found more suppression in the motor cortex after participants' viewed handwritten cursive letters compared to printed letters, suggesting that the motor cortex is recruited more by handwritten cursive letters. More recent studies also found recruitment of motor regions during perception of cursive letters. One study using Transcranial Magnetic Stimulation (TMS), found that recognition of handwritten cursive letters involved the motor cortex, as evidenced by reduced corticospinal excitability for the right hand (Nakatsuka et al., 2012). Another study found that Exner's area in the left dorsal premotor cortex was sensitive to whether dynamic cursive letters were presented forward or backward (Nakamura et al., 2012). To date only one fMRI study has looked at the neural correlates of perceiving handwritten cursive letters throughout the whole brain. Longcamp et al. (2011) replicated their previous results from the MEG study (Longcamp et al., 2006b) and found greater motor activation in the left primary motor cortex and supplementary motor area during the perception of handwritten cursive letters compared to the perception of hand-printed letters. Other non-motor areas were also implicated in the network for cursive letter processing, including the right superior frontal, middle occipital, and parahippocampal gyri, and the left inferior precentral and fusiform gyri. To date this is the only study that used neuroimaging methods at the whole-brain level to study the distinction between perceiving cursive and printed letters.

The recently uncovered neural networks for cursive letters in the adult brain implicate the motor cortex as a major part of the distinction seen in processing printed and cursive letters (Longcamp et al., 2006b, 2011; Nakatsuka et al., 2012; Nakamura et al., 2012). Although letter perception recruits the left motor cortex, perceiving cursive letters appears to enhance this activation in adults. To further understand why cursive letters have this effect, studying the *formation* of the neural substrates that underlie cursive writing would be fruitful. One purpose of the present work was to do just this: examine how networks are created during the initial experience of learning to write in cursive script. The question was whether very limited experience with writing in cursive would serve to link the motor and visual systems during letter perception or would extensive experience be required. That is, would we see the neural differences between manuscript print and cursive script that is observed in the adult, in children who are just learning to write in cursive script.

A second purpose of the present work was to investigate whether the motor act of writing letters was required for sensori-motor systems to respond to letter perception, or if observing another person write would result in the same neural response during subsequent perception. By introducing a true passive control in which the visual feedback of how the letter unfolds matches the visual feedback received during the production of letters, this study is the first to further isolate the effects of motor experience on the development of visual processing of letters. While previous research (James and Atwood, 2009; James, 2010; James and Engelhardt, 2012) suggests that the letters that children learn by motor production will develop functional specialization, it is unclear how perceptual networks will develop in response to letters learned in this true passive condition (where the visual “unfolding” of the percept is shown to the participants). If the act of motor production leads to functional specialization for letters, the letters that children learn by production would show a more adult-like response during subsequent perception than other cursive letters that the children do not produce themselves. However, if the visual feedback from motor production drives the specialization, then all learned cursive letters should show the same patterns of activation, whether actively produced or passively observed.

## Methods

### Participants

Seventeen right-handed children (6.9–7.8 years, mean age of 7.4 years, 9 female) participated in this study. Handedness was assessed using the Edinburgh Handedness Inventory (Oldfield, 1971). All children could read at or above a normal level for their age, print their name and selected letters, and had not yet started learning cursive in school. Additionally, all children had normal or corrected to normal vision and were native English speakers. Informed written consent was obtained from the parents who were compensated with a gift card, while the children were compensated with a small toy or book. All research was approved by the Indiana University Protection of Human Participants board.

### Materials

#### Behavioral training

**Figure 1:**
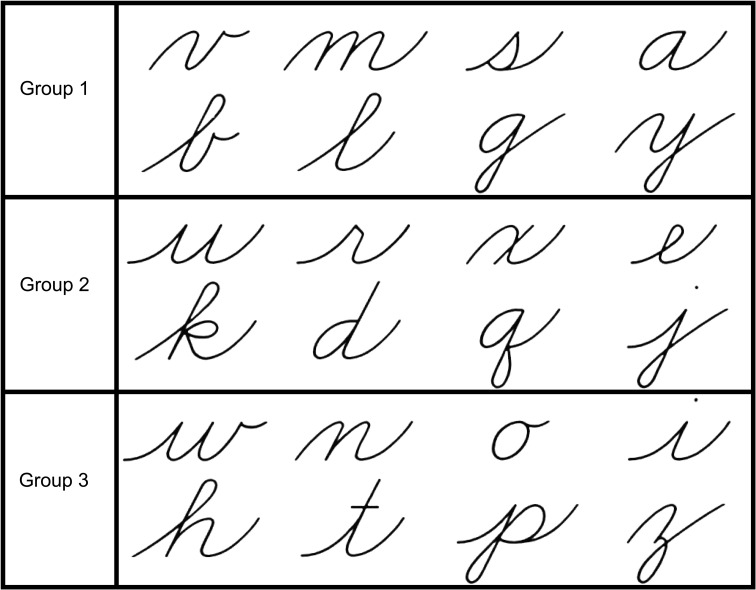
**Cursive stimuli divided into groups used for assignment of letters to learning conditions**.

**Figure 2:**
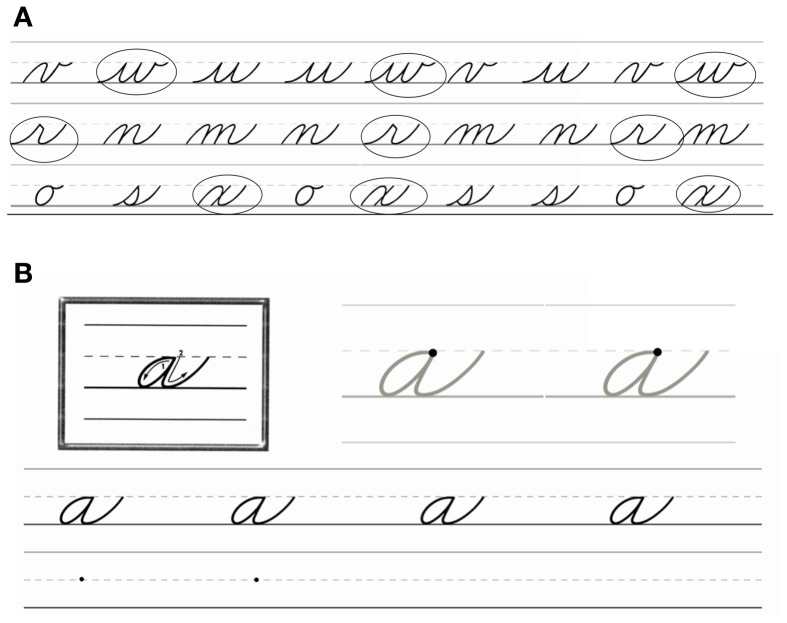
**(A)** An example of the cursive letter pre-test: participants circle each instance of a letter given to them. **(B)** An example of the worksheets used for learning to write cursive script.

Participants were taught to write letters in a cursive script that were from the Zaner-Bloser (ZB) script library and included twenty-four letters that were divided into three groups (see Figure 1). The letter “f” was excluded as it is the only letter that extends from below the writing line to the top of the writing space, and the letter “c” was excluded due to its similarity to the lowercase printed “c.” The cursive letter recognition test was created with ZB FontsOnline Plus and consisted of eight rows of cursive letters. Each row contained three similar letters, one from each set of cursive letters, repeated three times throughout the line (see Figure 2A for example). The recognition test was double-sided, resulting in a total of 16 rows so that each of the 16 learned letters could be tested.

In the cursive training session, each child learned one group of letters by motor production (actively) and one group by motor observation (passively), leaving one group of cursive letters unlearned. Training worksheets were modeled from worksheets in the Zaner-Bloser Handwriting Level 2C Practice Masters and created with the help of Zaner-Bloser's ZB FontsOnline Plus feature. The worksheets consisted of an example letter with arrows to demonstrate the order of the strokes in each letter, two large letters for tracing, and two sets of a pair of rows with the top row depicting each letter four times and the bottom row blank for children to write each letter under the model letter in the top row. In the bottom row of the first set, two starting dots were included to remind children where to start the letters. This resulted in each letter being traced twice and written eight times (see Figure 2B).

#### Imaging stimuli

Three groups of cursive letters were used as stimuli as well as printed letters. The printed letters were presented in Zaner-Bloser manuscript font and the cursive letters were presented in Zaner-Bloser cursive font (see Figure 1 for examples). All letters were presented in lower-case only. Each stimulus was presented individually and was 3” high by 2” wide. They were centered on the screen, and were presented in black font in a white box on a gray background to minimize brightness.

### Procedure

#### Pre-scan session

Prior to neuroimaging, children completed standardized tests and a behavioral training session. First, the WRAT 4 (Wide Range Achievement Test 4) (Wilkinson and Robertson, 2006) Word Reading Subtest Parts 1 (Letter Reading) and 2 (Word Reading) were administered to assess reading level. Part 1 (Letter Writing) of the WRAT4 Spelling Subtest was then administered to evaluate basic letter printing abilities. All children scored at or above normal for their age on all subtests. A pretest of cursive letter recognition was then administered in which children were asked to circle one letter per line when given the name of the letter (see Figure 2A). Children were tested on all cursive letters they were about to learn. Following the pretest, children learned eight letters actively by completing worksheets in which the strokes required to write the letters were described as the experimenter pointed to reference points on the example letter. They then traced each letter twice and wrote each letter eight times (see Figure 2B). Children learned eight additional letters passively by watching an experimenter complete a worksheet in the same format, matching for time to ensure approximate equal exposure to the letters learned actively and passively. The child and experimenter alternated learning letters and after each had taken a turn, the child was allowed to select a sticker. This encouraged children to pay attention while the experimenter completed her turn. The active and passive sets of letters learned and the order in which the cursive letters were learned were counterbalanced across participants. After the training, and prior to neuroimaging, the same cursive letter recognition was administered as a posttest. The order of target letters for each line was counterbalanced between pre and posttests and across participants.

#### Imaging session

Immediately prior to the imaging session children were taken to an MR simulator so that they could acclimate to the MR environment. Children watched short cartoons while simulated sounds recorded from actual EPI sequences were played in the background. This ensured that participants were not surprised by the loud noises or the confined space. Children practiced lying still while head and body movements were monitored. If they felt comfortable in this environment and if both the child and their parent consented to continuing with the actual neuroimaging, they were then taken directly to the MR scanner.

At the beginning of the imaging session, a preliminary high-resolution anatomical scan was administered while the child watched a cartoon. Following the anatomical scan were four functional runs. Children were instructed to simply look at the stimuli as they appeared (passive viewing). Stimuli were presented via SuperLab Pro 4 (Cedrus Corporation) on a Macintosh Macbook laptop. A block design was used. Each block consisted of 8 stimuli and lasted for 16 s with 12 s of fixation between each block and a 20 s period of fixation at the beginning of each run. Each stimulus block was repeated at least once per run so that across the 4 runs each stimulus type was presented 5 times. This resulted in two longer runs of 8 blocks lasting 4 min 4 s and two shorter runs of 7 blocks lasting 3 min 36 s. The longer runs were always administered first and the shorter runs followed. Note that there were 2 additional stimulus conditions presented that are not analyzed here, consisting of shape stimuli and were included for another study. Neural activation during perception of the different stimuli was measured by the blood oxygen-level-dependent (BOLD) signal throughout the brain. Imaging sessions lasted approximately 30 min.

### fMRI acquisition

Imaging was performed using a 3-T Siemens Magnetom Trio whole-body MRI system and a phased-array 12-channel head coil, located at the Indiana University Department of Psychological and Brain Sciences. Images were acquired using an echo-planar technique (*TE* = 20 ms, *TR* = 2000 ms, flip angle = 90°) for BOLD-based imaging. The field of view was 22 × 22 × 9.9 cm, with an in-plane resolution of 64 × 64 and 33 slices per volume that were 4 mm thick. The resulting voxel size was 3.0 × 3.0 × 4.0 mm. Functional data underwent slice-time correction, 3D motion correction, linear trend removal, and Gaussian spatial blurring (FWHM 6 mm) using the analysis tools in Brain Voyager™. Individual functional volumes were co-registered to anatomical volumes with an intensity-matching, rigid-body transformation algorithm. Co-registration was to the anatomical volumes acquired at the beginning of the runs. Voxel size of the functional volumes was standardized at 1 × 1 × 1 mm using trilinear interpolation.

### fMRI data analysis procedures

Whole-brain group contrasts were performed on the resulting data. The functional data were analyzed with a random effects general linear model (GLM) using the Brain Voyager™ multi-subject GLM procedure. The GLM analysis allows for the correlation of predictor variables or functions with the recorded activation data (criterion variables) across scans. The predictor functions were based on the blocked stimulus presentation paradigm of the particular run being analyzed and represent an estimate of the predicted hemodynamic response during that run. Only functional data from right-handed children were analyzed. Any functional data that exceeded 5 mm of motion on any axis during a block other than fixation was excluded from the analysis. This resulted in the exclusion of data from 9 right-handed participants consisting of 3 volumes of data from actively learned letters, 6 volumes from passively learned letters, 4 volumes from unlearned cursive letters, and 2 full runs from one participant. Additionally, one right-handed participant did not complete the MR imaging session. This resulted in data from 16 right-handed children. Individual anatomical data was normalized to the stereotactic space of Talaraich and Tournoux (1988) using an eight-parameter affine non-linear transformation, with parameters selected by visual inspection of anatomical landmarks and by using the Talairach Deamon Applet and Client (Lancaster et al., 1997, 2000).

## Results

### Overt learning performance

Overall cursive recognition performance prior to training was 56% (SE.05) and post-training recognition was at 80% (SE.03). To better understand the recognition performance the cursive recognition tests were scored for the 16 participants (that completed the MRI scanning) using a sensitivity measure. When the child circled the target letter, a hit was scored, and when a distractor letter was circled, a false alarm was recorded. Probability of a hit and probability of a false alarm were then calculated for each test. The probability of a false alarm was subtracted from the probability of a hit to obtain a measure of sensitivity in distinguishing amongst the cursive letters. This measure of sensitivity was used in all statistical tests. A 2 (time: pre-learning and post-learning) × 2 (learning condition: active vs. passive) repeated measures ANOVA was performed and revealed only a main effect of time [*F*_(1, 15)_ = 19.30, *p* < 0.001], but no main effect of learning [*F*_(1, 15)_ = 2.1, n.s.] or an interaction [*F*_(1, 15)_ = 0.35, n.s.]. Thus, there was a learning effect (change over time), but this was not specific to the learning condition.

**Figure 3:**
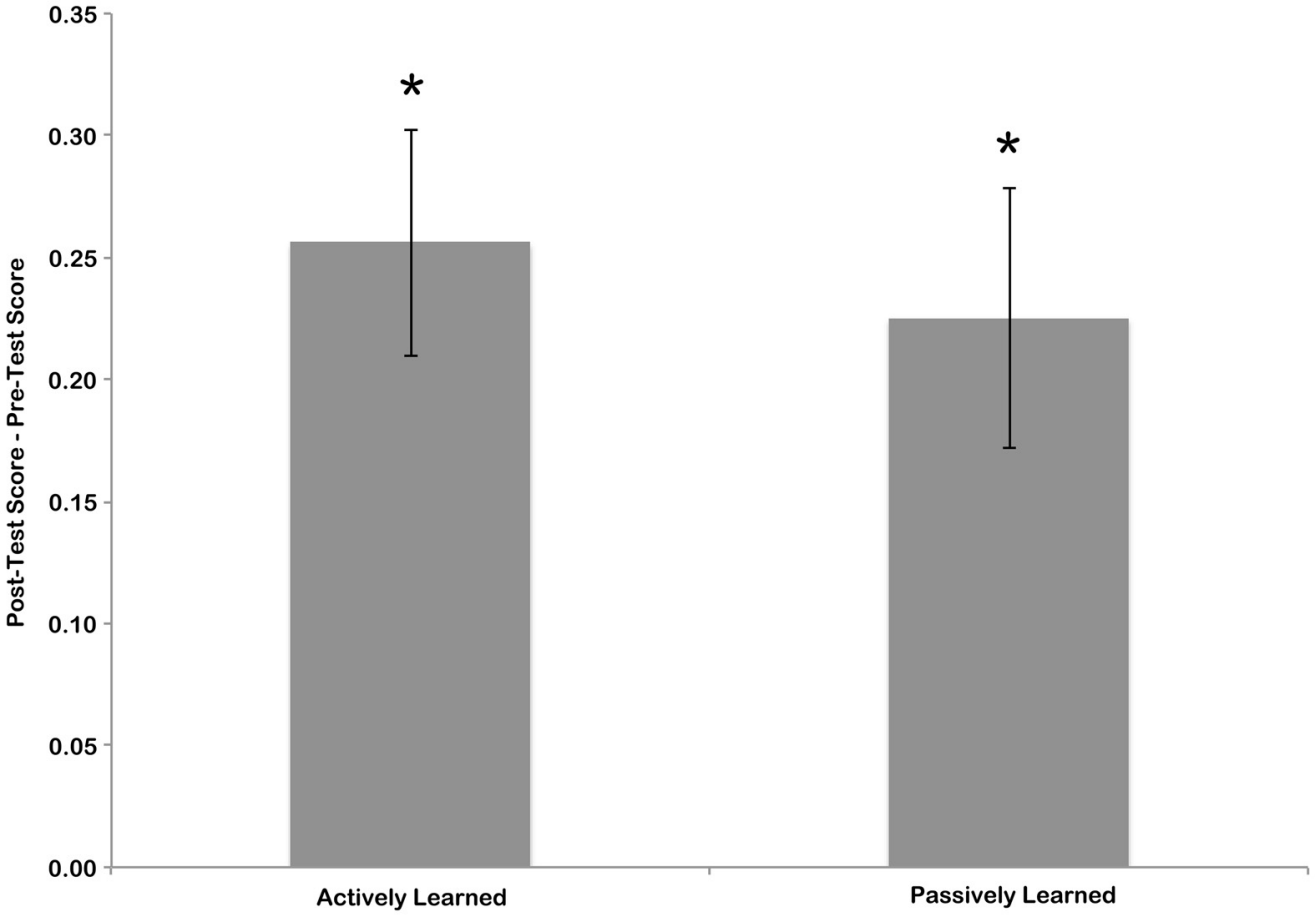
**Difference in pre-test and post-test scores for actively and passively learned letters.** Error bars are standard error of the mean, and asterisk indicate significant difference from chance (no learning) at *p* < 0.05.

To measure the amount of learning that took place during training, the sensitivity score for the pre-test was subtracted from the sensitivity score of the post-test. This was done separately for the letters that the children learned actively, by writing, and the letters that children learned passively, by watching an experimenter write (see Figure 3). To determine if significant learning took place these scores were compared to zero (no learning) using one-sample *t*-tests. *T*-tests indicated that the change in scores from pre-test to post-test was significant for both actively learned letters [mean = 0.2467, *t*_(15)_ = 5.13, *p* < 0.01] and passively learned letters [mean = 0.2145, *t*_(15)_ = 4.12, *p* < 0.01]. A paired samples *t*-test revealed no difference in amount of learning between the active and passive conditions [*t*_(15)_ = 0.698, n.s.]. This suggests that although children were better at identifying cursive letters after the training session, each learning condition resulted in the same amount of learning.

### Neural activation

#### Whole-brain analyses

Several whole-brain contrasts were performed. Individual data was averaged together to create group statistical parametric maps. All results are reported at a voxel-wise error-rate of *p* < 0.01 with a cluster-threshold correction of 8 voxels to obtain an overall family-wise error rate of *p* < 0.05. Reported trends are for areas of activity significant at a voxel-wise error rate of *p* < 0.01 with no cluster-threshold correction applied. See Table 1 for a complete list of brain regions recruited for the following contrasts.

**Table 1:** **Peak Talairach coordinates and range of distribution for each region revealed in whole brain contrasts**.

**Contrast**	**Region**	**Talairach Peak (*X, Y, Z*)**	***X*-range**	***Y*-range**	***Z*-range**	**Total voxel size**
Print vs. actively learned	Right superior temporal gyrus	31, 15, −23	23… 37	2… 20	−26… −17	560
	Right parahippocampal gyrus	25, −2, −18	7… 33	−14… 2	−23… −9	956
	Right cuneus	23, −84, 32	19… 29	−87… −79	29… 40	261
	Left superior parietal lobule	−24, −53, 61	−31… −20	−54… −47	57… 66	716
		−18, −49, 63	−24… −12	−56… −42	56… 70	830
	Left medial frontal gyrus	−5, −26, 67	−17… 1	−39… −21	59… 76	1546
	Left postcentral gyrus	−6. −39, 66	−11… 1	−44… −35	57… 72	945
		−14, −34, 67	−19… −10	−36… −27	65… 72	423
	Left precentral gyrus	−37, −26, 62	−47… −28	−35… −21	56… 69	895
	Left precuneus	−4, −49, 62	−12… 2	−61… −45	54… 70	1598
		2, −59, 49	−8… 11	−80… −53	39… 57	963
		−30, −80, 36	−38… −7	−89… −73	29… 50	1095
	Left cuneus	−1, −95, 15	−16… 4	−102… −86	0… 22	956
	Left cerebellum	−11, −71, −14	−16… 5	−73… −65	−15… −10	292
Print vs. passively learned	Right insula	34, 6, 13	27… 40	3… 14	12… 17	395
	Right precentral gyrus	49, −15, 29	45… 54	−17… −12	24… 33	230
	Right cuneus	4, −91, 22	0… 21	−95… −85	16… 36	536
	Right cerebellum	25, −37, −18	20… 29	−49… −31	−20… −15	294
		23, −61, −11	19… 32	−69… −29	−16… −7	604
		9, −60, −12	−2… 22	−66… −43	−16… −7	2151
	Left precentral gyrus	−62, 0, 7	−68… −52	−9… 11	−3… 19	2079
		−48, −12, 45	−54… −42	−24… −6	37… 61	1148
		−35, −14, 51	−40… −32	−16… −9	45… 58	263
	Left inferior parietal lobule	−63, −32, 32	−68… −56	−42… −22	20… 40	1093
		−60, −29, 24	−65… −52	−24… −14	24… 30	487
	Left postcentral gyrus	−42, −25, 67	−46… −37	−29… −21	53… 61	274
	Left cerebellum	−25, −32, −20	−30… −18	−37… −27	−23… −16	451
	Left cerebellum	−3, −81, −12	−18… 15	−93… −80	−17… 2	1352
Print vs. unlearned	Right superior frontal gyrus	−35, 43, 31	−39… −27	39… 51	27… 37	562
	Right superior frontal gyrus	−35, 43, 31	19… 27	43… 52	30… 42	418
	Right insula	38, 8, 11	33… 42	2… 11	8… 13	226
		49, −18, 22	44… 55	−25… −13	17… 31	825
	Right precentral gyrus	48, −8, 9	39… 60	−14… 0	6… 15	819
	Right postcentral gyrus	17, −42, 66	11… 26	−50… −37	63… 70	680
	Right precuneus	4, −61, 57	−12… 20	−69… −42	50… 69	1970
	Right caudate	35, −42, 9	31… 43	−48… −40	2… 15	682
	Right lingual gyrus	29, −57, −2	26… 34	−69… −47	−11… 2	351
	Right cerebellum	27, −49, −16	21… 33	−54… −42	−21… −12	409
	Left putamen	−28, −9, −7	−32… −21	−15… −7	−13… −2	255
	Left transverse temporal gyrus	−45, −28, 13	−56… −36	−38… −25	5… 22	491
	Left precentral gyrus	−15, −24, 67	−24… −7	−35… −15	62… 73	1227
	Left postcentral gyrus	−29, −37, 62	−37… −22	−34… −20	59… 67	937
	Left superior parietal lobule	−21, −61, 56	−40… −10	−71… −42	47… 67	2159
	Left cerebellum	−38, −47, −17	−43… −33	−56… −38	−21… −9	721
Actively learned vs. passively learned	Right claustrum	30, 10, 15	27… 34	5… 18	13… 17	143
	Right insula	38, 3, 13	33… 43	1… 10	11… 17	212
	Left precentral/postcentral gyri	−50, −11, 46	−55… −49	−12… −5	44… 49	88 n/s
	Left claustrum	−37, −13, −4	−40… −32	−22… 4	−9… 7	1110
		−34, −2, 10	−43… −27	−6… 14	5… 17	790
All cursive vs. rest						
	Right fusiform gyrus	41, −71, −8	34… 47	−88… −64	−18… −3	1138
		28, −92, −10	22… 34	−99… −88	−14… −4	500
	Left middle ociipital gyrus	−46, −77, −10	−52… −17	−102… −50	−24… 2	5359
Actively learned vs. rest						
	Right fusiform gyrus	40, −43, −9	36… 47	−55… −37	−14… −2	1127
	Right cerebellum	35, −42, −21	30… 39	−45… −37	−26… −17	255
	Left fusiform gyrus	−40, −53, −13	−43… −37	−57… −47	−19… −10	266
	Left cerebellum	−40, −38, −21	−46… −31	−47… −26	−29… −17	909
	Posterior cingulate	1, −30, 23	−10… 9	−36… −20	17… 27	1178
Unlearned vs. rest	Left cerebellum	−45, −72, −16	−51… −39	−83… −65	−21… −8	1237
	Left Inferior occipital gyrus	−45, −78, −4	−49… −41	−84… −71	−9… 0	539

**Figure 4:**
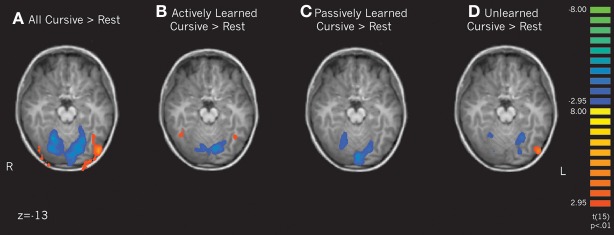
**Contrasts of cursive letters > rest focusing on the lateral occipital complex and the fusiform gyrus.** Depicts individual contrasts of **(A)** all cursive letters > rest, **(B)** actively learned cursive letters > rest, **(C)** passively learned cursive letters > rest, and **(D)** unlearned cursive letters > rest. Family-wise error rate held at *p* < 0.01.

***Letter perception vs. rest***. To determine how learning cursive letters actively and passively affected subsequent perception, we performed several contrasts of our learning conditions. First, we compared the perception of all cursive letters to rest. This analysis revealed that when comparing all cursive letters to rest, an extensive region in the Lateral Occipital complex, including the fusiform gyrus, was active bilaterally (see Figure 4A). When comparing the individual learning conditions to rest, only the actively learned cursive letters recruited this region, and only in the fusiform gyrus, more than rest (Figure 4B). Passively learned letters did not recruit this region greater than rest (Figure 4C). Therefore, just as is seen with printed letters, perceiving cursive letters only recruits letter-specific processing regions after active learning. We also compared unlearned cursive letters to rest, which recruited the left LOC, but not the fusiform gyrus (see Figure 4D). No regions were significantly active in the remainder of the brain above rest, including motor cortex.

***Cursive perception vs. print perception***. Three contrasts were performed in an attempt to replicate the findings from Longcamp et al. (2011) of neural differences in processing cursive letters vs. letters printed in manuscript form. All cursive letters (actively learned, passively learned, and unlearned) were compared against printed letters to determine how different learning experiences affect the perception of cursive letters in relation to familiar printed ones. This contrast was balanced by weighting the printed condition × 3. Unlike the previous findings, cursive letters were not found to significantly recruit any regions more so than printed letters. However, the reverse contrasts revealed several areas that were recruited more for printed than cursive letters. We unpacked these differences by then contrasting each individual cursive learning type (active, passive, and unlearned) to printed letters.

**Figure 5:**
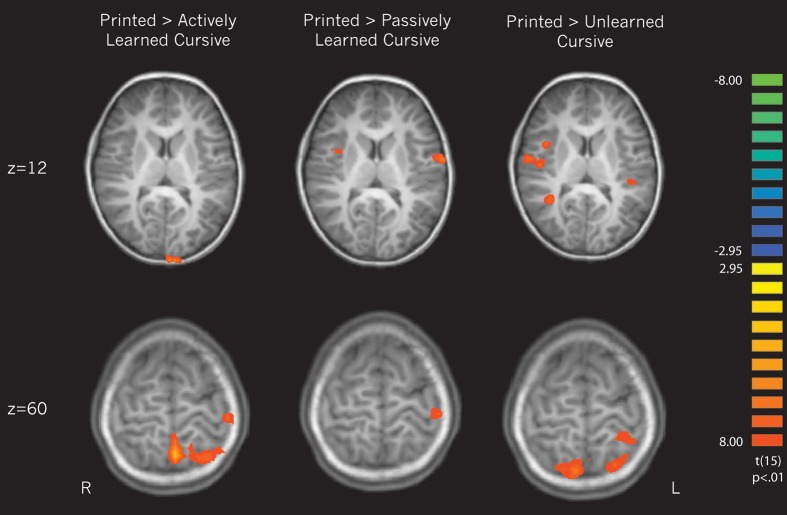
**Direct contrasts of perception of printed letters > perception of cursive letters broken down by cursive learning condition.** The upper slice denotes of pattern of interest regarding the right insula, which is recruited when printed letters are compared only to passively learned and unlearned cursive. The bottom slice reveals greater recruitment of motor regions for printed letters in comparison to each type of cursive letter.

- Contrasting printed letters with *actively* learned cursive letters revealed significant activity in the right superior temporal gyrus, right parahippocampal gyrus, the left precentral gyrus, the left medial frontal gyrus, left cerebellum, and several parietal areas including the left postcentral gyrus and left superior parietal lobule, indicating that those areas are recruited more for printed letters in comparison to actively learned cursive letters (see Figure 5, left).
- In comparing the printed letters with *passively* learned cursive letters, printed letters were found to recruit the right superior temporal gyrus, right insula, bilateral precentral gyrus, left postcentral gyrus, bilateral cerebellum and bilateral parahippocampal regions more than for passively learned cursive letters (see Figure 5, middle).
- Finally, in comparison to *unlearned* cursive letters, the printed letters recruited the bilateral superior frontal gyrus, the right insula, the left and right precentral gyrus, the left putamen, the right transverse temporal gyrus, bilateral postcentral gyrus, and the cerebellum to a significantly greater degree (see Figure 5, right). In sum, these results indicate that printed letter perception recruited several regions more than perception of cursive letters. Furthermore, the location and extent of the recruitment was different when comparing perception of printed letters to each of the three groups of cursive letters, indicating an effect of learning. Of particular note is that the right insula was not recruited differentially to printed and actively learned cursive letters, but was for printed letters vs. passive and unlearned cursive letters.

Combined with the cursive vs. rest contrasts, these results indicate that although active learning does result in the recruitment of the fusiform gyrus (active cursive vs. rest) during cursive letter perception, it does not do so more than the perception of printed letters.

**Figure 6:**
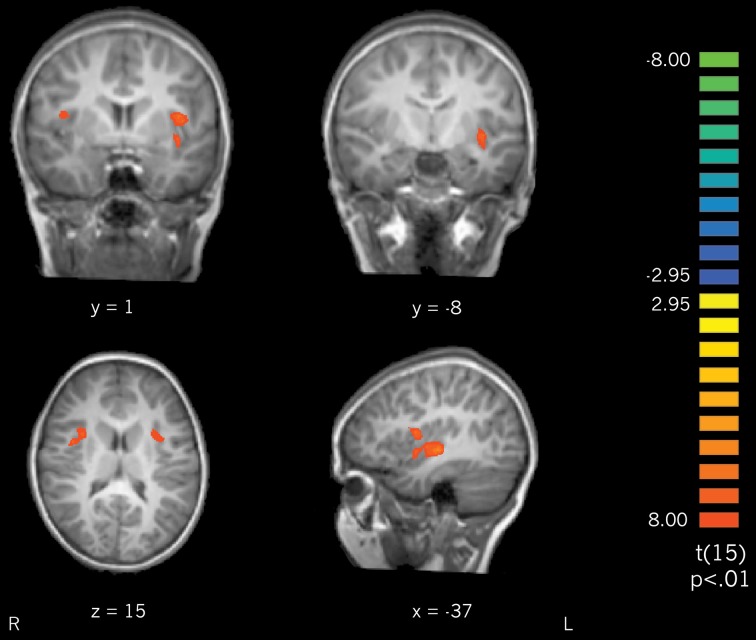
**A contrast of perception of actively learned letters > passively learned letters resulted in recruitment of bilateral insula and claustrum**.

***Actively learned vs. passively learned cursive letters***. To investigate the differences between learning conditions directly, actively learned and passively learned cursive letters were compared. Contrasting the actively learned cursive letters with the passively learned cursive letters revealed significant recruitment of the bilateral insula and claustrum for the actively learned letters (Figure 6) with trends toward significantly greater recruitment of the left precentral and postcentral gyri (not pictured). These results indicate that self-generated writing leads to greater recruitment of the insula and claustrum regions during subsequent letter perception than does observing letter writing. Interestingly, active vs. passive learning did not differ in the fusiform gyrus when contrasted directly, suggesting that the difference in activation outlined above, when these conditions are compared to rest, is a small (but significant) effect.

#### Region of interest analyses

The following analyses were performed on children who showed recruitment of the left fusiform gyrus in a contrast of printed letters > rest at the same threshold used in the whole-brain analyses (voxel-wise error-rate of *p* < 0.01 with a cluster-threshold correction of 8 voxels). This restriction ensured that the following analyses included only those children who were responding to printed letters in an adult-like manner and resulted in the exclusion of four children, leading to a sample size of 12. See Table 2 for a complete list of each individual's regions of interest. Note that the peak activity resulting from this contrast did not result in true fusiform activity (as determined by the Talaiach Daemon) in each individual. Nonetheless, we extracted data from these regions for the following reasons: (A) in each individual the peak of activity fell within 5 mm of the fusiform gyrus (as determined by the Talairach Client); (B) given this is functional localization, these regions responded more to print than rest and were within 5 mm of the fusiform gyrus and (C) given co-registration difficulties with children's brain on an adult template, and that visually, the regions appeared to lie within the cortex, we believe that using a 5 mm range is appropriate.

**Table 2:** **Peak Talairach coordinates and range for the Regions-of-Interest analyses**.

**ROI**	**Participant**	**Talaraich Peak (*X, Y, Z*)**	***X*-range**	***Y*-range**	***Z*-range**	**Total voxel size**
**Left fusiform region (+/− 5 mm)**	AT	−39, −61, −18	−44… −36	−65… −58	−21… −13	212
	ES	−43, −50, −20	−46… −41	−56… −47	−23… −17	117
	GM	−30, −46, −17	−33… −28	−51… −43	−21… −14	160
	HG	−39, −62, −15	−43… −35	−65… −60	−19… −12	273
	JM	−41, −47, −15	−43… −39	−51… −44	−20… −12	222
	KP	−38, −65, −10	−42… −34	−70… −61	−15… −6	516
	LS	−36, −54, −14	−37… −33	−59… −51	−18… −12	112
	MK	−41, −59, −19	−44… −39	−62… −54	−22… −17	154
	MP	−48, −42, −23	−52… − 43	−44… −38	−28… −19	438
	OS	−45, −52, −19	−49… −40	−57… −48	−24… −15	474
	SL	−38, −50, −18	−41… −34	−55… −46	−23… −14	314
	SM	−43, −48, −12	−46… −40	−53… −44	−15… −9	255
**Right fusiform region (+/− 5 mm)**	AT	39, −66, −15	35… 42	−69… −63	−18… −12	230
	ES	31, −58, −18	28… 34	−60… −54	−22… −15	148
	GM	21, −45, −17	18… 27	−48… −41	−20… −14	216
	HG	30, −53, −5	28… 34	−59… −50	−8… −3	154
	JM	36, −48, −22	33… 38	−52… −45	−24… −20	115
	KP	37, −64, −9	34… 40	−68… −61	−11… −8	102
	LS	36, −49, −15	30… 39	−52… −43	−19… −11	285
	MK	33, −48, −11	30… 35	−52… −43	−15… −8	140
	MP	47, −41, −19	41… 49	−46… −37	−22… −15	184
	OS	34, −44, −20	30… 39	−49… −41	−24… −17	367
	SL	36, −38, −17	31… 40	−43… −34	−23… −14	420
	SM	44, −58, −8	40… 48	−63… −54	−14… −5	272
**Precentral gyrus**	AT	−47, −14, 41	−51… −42	−17… −11	38… 46	261
	ES	−49, −9, 56	−53… −46	−12… −6	53… 58	67
	GM	−48, −3, 53	−51… −45	−6… 0	50… 55	46
	HG	−57, −5, 42	−60… −54	−8… −2	37… 46	131
	JM	−44, −7, 52	−49… −40	−9… −5	50… 55	167
	KP	−46, −6, 41	−49… −43	−8… −3	39… 44	61
	LS	−48, −8, 49	−52… −44	−12… −4	47… 51	118
	MK	−34, −9, 62	−39… −31	−13… −6	58… 65	208
	MP	−57, −4, 44	−60… −54	−7… 0	41… 47	131
	OS	−47, −13, 41	−49… −44	−15… −11	38… 43	89
	SL	−24, −14, 47	−27… −22	−15… −11	45… 49	39
	SM	−29, −9, 49	−34… −25	−11… −7	46… 52	104

**Figure 7:**
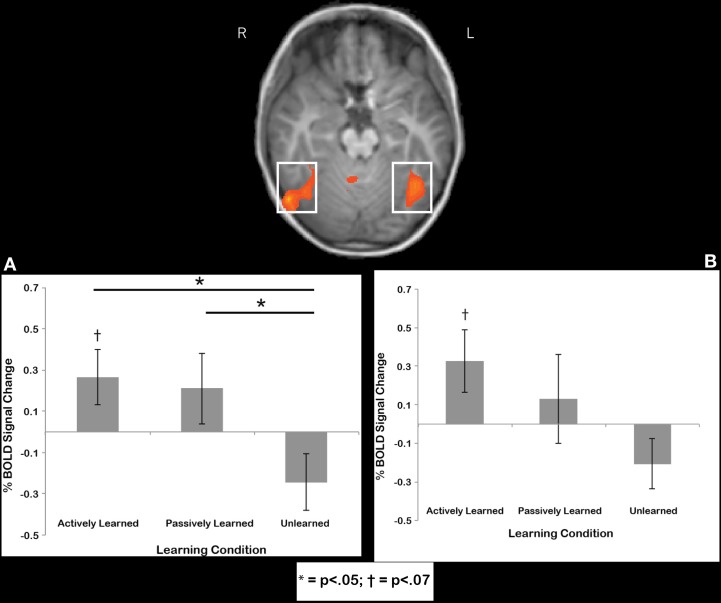
**Region of interest results for the right fusiform (A) and left fusiform (B).** Trends were found for recruitment of the left and right fusiform above baseline in response to perception of actively learned letters. Significant differences in recruitment for perception of actively learned letters and passively learned letters compared to unlearned cursive letters were found in the right fusiform only. Error bars are standard error of the mean.

***Fusiform gyrus region***. Regions-of-interest were determined by comparing printed letters to rest in a whole-brain comparison in each individual. This contrast was used to reveal the well-known brain regions that respond to letters after a child learns to print, in particular, the bilateral anterior fusiform gyrus (e.g., James, 2010; James and Engelhardt, 2012), located at the peak coordinates of −39, −54, −15 and 38, −54, −15. We extracted data from this region that is independent of the contrast used to select the region. Our whole-brain contrasts suggested that there should be an active cursive vs. passive cursive difference in this region, but it is probably a small effect, thus, the ROI analysis can show us a direct comparison with more statistical power. To determine whether or not the cursive letters recruited these regions, we extracted data from individuals from these two ROIs and performed statistical tests on the mean time courses of BOLD activation for the cursive letter conditions. First, we wanted to determine whether the signal in these regions was significantly above baseline, and therefore performed one-sample *t*-tests of each learning condition (active, passive unlearned) against baseline. Results indicated a trend toward significant recruitment above baseline for actively learned letters in the left fusiform gyrus [*t*_(11)_ = 2.02, *p* = 068] (Figure 7A) and right fusiform gyrus [*t*_(11)_ = 1.98, *p* = 0.073], (Figure 7B) but no other contrasts approached significance. Next, to compare how learning condition affected % BOLD signal change, a 2 (hemisphere) X 3 (Learning condition: active, passive, unlearned) mixed model ANOVA was performed that revealed no significant differences in terms of main effects or interactions, but two interesting trends: One for Learning condition [*F*_(2, 11)_ = 2.7, *p* = 0.09], and another for the Learning X hemisphere interaction [*F*_(2, 22)_ = 2.6, *p* = 0.09]. Keeping in mind the small sample size, we chose to further investigate these trends by performing two One-Way ANOVAs for each hemisphere separately. No effect of learning condition was found in the left fusiform gyrus [*F*_(2, 11)_ = 2.078, n.s.]. However, an effect of learning condition was found in the right fusiform gyrus [*F*_(2, 11)_ = 3.435, *p* < 0.05]. *Post-hoc* paired *t*-tests revealed a significant difference between the actively learned letters and the unlearned letters [*t*_(11)_ = 2.369, *p* < 0.05, mean difference of 0.51, SD = 0.75) and between the passively learned letters and the unlearned letters [*t*_(11)_ = 2.511, *p* < 0.05, mean difference of 0.45, SD = 0.64]. No significant difference was found between actively learned letters and passively learned letters [*t*_(11)_ = 2.34, n.s., mean difference of 0.06, *SD* = 0.83] (see Figure 7A). In sum, writing letters trended toward significant recruitment of the bilateral fusiform above baseline, while both self-generated active and observed passive writing led to greater recruitment of the right fusiform during subsequent perception in comparison to perception of unlearned letters.

**Figure 8:**
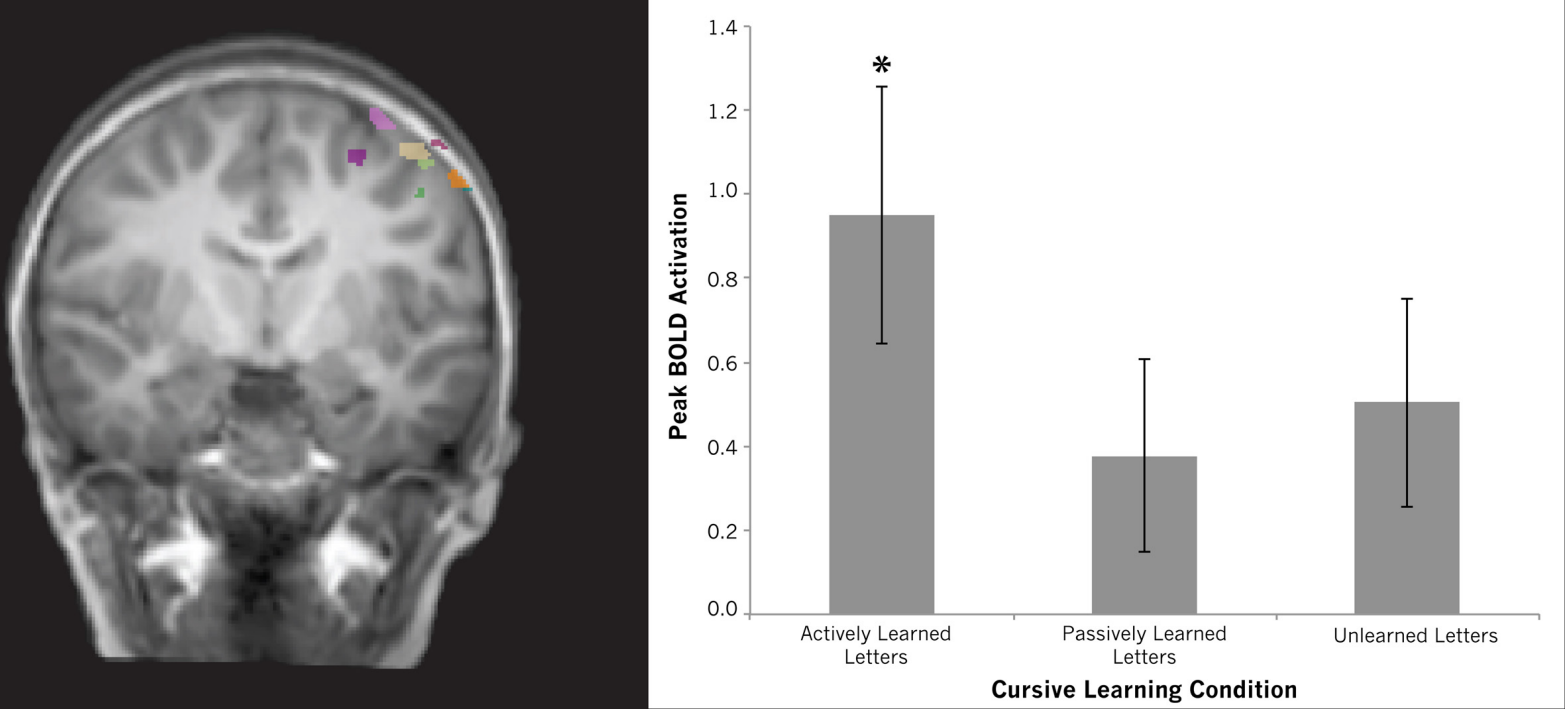
**Region of interest results for the left motor cortex.** Brain map shows individual regions-of-interest superimposed on a typical brain. Graph shows differences among conditions extracted from these regions. Although no differences were found between conditions, only viewing actively learned letters recruited this region significantly above baseline (indicated by asterisk). Error bars are standard error of the mean.

***Motor cortex***. Our whole brain contrasts suggested that the left precentral gyrus may be differentially recruited for actively learned vs. passively learned cursive letters as indicated by a trend toward greater recruitment of this region during perception of actively learned letters compared to passively learned ones. This ROI analysis was designed to further investigate this difference. Regions-of-Interest within the motor cortex were identified by contrasting printed letters with rest in a whole-brain comparison for each individual. This resulted in a region-of-interest with peak coordinates of −41, −16, 59, situated in the left precentral gyrus. All peak coordinates from each individual fell on the left precentral gyrus (see Table 2). To determine how this region responded to the perception of cursive letters after different learning conditions, a One-Way repeated measures ANOVA was performed using each individual's peak motor cortex activation. The ANOVA revealed no effect of learning condition [*F*_(2, 11)_ = 1.093, n.s.]. Planned comparisons were performed to investigate whether any of the learning conditions recruited this region above baseline. One-sample *t*-tests were performed for each learning condition, resulting in a significant difference for actively learned letters from baseline [mean = 0.951, *t*_(11)_ = 3.102, *p* > 0.01], but no effect for passively learned letters [mean = 0.378, *t*_(11)_ = 1.645, n.s.] or unlearned cursive letters compared to baseline [mean = 0.503, *t*_(11)_ = 2.035, n.s.] (see Figure 8). This strengthens our assumption that active learning of letters drives the recruitment of the precentral gyrus during letter perception.

## Discussion

This study investigated the role of motor experience in the development of neural networks for cursive letters. Behavioral measures of recognition and neural measures of perception were taken to evaluate how cursive letters were processed after learning relative to printed letters and how active (self-generated) and passive (observed) motor experience influenced these processes. There were several results of interest: First, viewing cursive letters recruited the bilateral fusiform gyrus only when the letters were learned through self-generated action. This finding was shown by our group contrasts (active vs. rest) and by the ROI analysis of the fusiform gyrus. Next, the precentral gyrus was also recruited more for actively learned letters compared to passive observation. Further, the bilateral insula and claustrum were recruited during perception of the letters that children wrote, compared to letters that children observed being written.

Interestingly, no regions were found to be recruited more for cursive letters in comparison to printed ones. This null result is interesting in that it fails to replicate a finding for adults (Longcamp et al., 2011) suggesting that the amount of learning in this study was not sufficient to dissociate the perception of cursive letters from printed letters.

Together, these results suggest that (A) active learning of cursive letters recruits the same sensori-motor regions in the brain as do printed letters (James, 2010; James and Engelhardt, 2012); (B) that active learning of cursive letters also recruits additional brain areas more than passive learning of the same stimuli; and (C) that perceiving cursive letters in general does not recruit additional brain areas relative to perceiving printed letters. We will address these conclusions in more detail below.

### Behavioral results

Behavioral results indicated that children were better at discriminating among cursive letters after the learning session compared to before learning. This is not surprising given that children had no formal training in cursive letters prior to the study and is in line with other studies that found increased recognition abilities after learning a new written stimulus (Longcamp et al., 2005b; James and Atwood, 2009). However, learning did not differ based on learning condition, suggesting that the neural differences seen in the whole-brain and region-of-interest analyses are a result of training condition rather than superior performance in ability to recognize letters learned in one condition over another.

### fMRI results

#### Self-generated action and the sensori-motor network

***The fusiform gyrus***. Similar to perceiving printed letters, we show that perceiving cursive letters results in the recruitment of the fusiform gyrus, bilaterally. Further, this finding is more robust (being shown in both whole-brain and ROI analyses) if the letters are learned through self-generated action. This finding replicates and extends our previous work (James, 2010; James and Engelhardt, 2012) showing that active learning of print stimuli results in the recruitment of letter specific neural processing regions seen in the literate adult. Furthermore, this finding resulted from a very limited learning episode, similar to our findings in James and Engelhardt (2012). We interpret this result as indicating that motor experience with these stimuli enhances—or most certainly changes—subsequent visual processing. There are several reasons why this change may occur. First, motor movements may direct attention to the stimuli being learned, resulting in a more robust visual representation. Second, after a motor movement is learned, a motor program is established that may directly connect to the visual cortex during perception, increasing its recruitment. Third, the act of writing a letter may result in visual information that adds to, or augments, a representation of the visual form that is re-activated upon subsequent perception. The first hypothesis, that motor movements direct greater attention to visual processing, was addressed in previous research that showed that typing, and even tracing letters, did not result in the same sensori-motor recruitment as self-generated action—printing in that case (James and Engelhardt, 2012). It is still possible though, that self-generated action drives visual *attention* more than typing or tracing, but we find this interpretation to be somewhat unlikely. The second hypothesis, that the motor program established after writing directly contacts visual processing and serves to change visual-only processing seems also limited because of the finding that tracing (the same motor program) does not enhance visual processing to the same degree as self-generated writing (James and Engelhardt, 2012). The third hypothesis, and the one that we favor, is that the output from self-generated handwriting produces variable (messy) stimuli when children produce the form. This output is then processed by the visual system. The perception of the variable forms may serve to broaden perceptual categories, and in turn, enhance visual processing of that stimulus class. We have preliminary evidence that this is the case. When young children are asked to categorize a single form of letter-like stimuli (Greek symbols) vs. variable forms of these same stimuli, their subsequent categorization of the stimuli is facilitated if they practiced categorizing variable or messy, symbols (Li and James, 2013; in preparation). This behavioral evidence, however, does not address the neural hypothesis proposed here and further work is required to address this issue using neuroimaging methods. Perhaps stronger evidence comes from our passively learned letter condition, where experimenters produced the cursive letters for the children to see. In this case, the letters were produced in accordance to the typical cursive font, with little or no variability. Observing these letters did not result in recruitment of the sensori-motor network seen in this, and previous work. In line with this hypothesis are results from a recent study that found suppression in the visual-word form area and its right hemisphere homolog for repeated words written in the same font, suggesting a sensitivity to font in the fusiform (Barton et al., 2010). Because the letters produced in the passive learning condition were written in a consistent handwriting style, this consistency may have suppressed the fusiform during learning, thus inhibiting reactivation of this region above baseline during subsequent perception. In contrast, the actively learned letters were produced with more variability, preventing repetition-suppression from occurring during learning and permitting a more typical pattern of recruitment for letters within the fusiform. However, the idea of this type of sensitivity to font within the fusiform is relatively new and should be interpreted with caution.

Interestingly, comparing learning conditions to *each other* (rather than to baseline or rest) within the left and right fusiform led to differences between conditions only within the right fusiform. Learned letters were found to more strongly recruit this region in comparison to unlearned letters, but there was no difference found between actively and passively learned letters. The fusiform gyri have been implicated in the perception of several stimulus categories including letters, faces, and objects. However, hemispheric differences are typically found based on the particular type of stimulus. The right fusiform, which has been linked with the perception of objects associated with expertise, tends to be recruited for perception of cars and birds (Gauthier et al., 2000), faces (Puce et al., 1995; Kanwisher et al., 1997), and well-learned greebles (Gauthier et al., 1999). In contrast, the left fusiform is typically associated with perception of words (e.g., Joseph et al., 2003, 2006; Turkeltaub et al., 2003; Cohen and Dehaene, 2004; Schlaggar and McCandliss, 2007) and letters (e.g., James and Gauthier, 2006; James, 2010). Despite this traditional lateralization for object perception, the right fusiform has been implicated in letter perception shortly after learning, but only in addition to recruitment of the left fusiform (James and Atwood, 2009; James, 2010; James and Engelhardt, 2012). The hypothesis that early in learning systems are bilateral and then shift to unilateral organization with increased experience has been proposed by several theorists (Johnson, 2011; Plaut and Behrmann, 2011). Specifically, these theories suggest that the left hemisphere preference for processing words, and perhaps even for letters, is driven by learning to read, which enhances the connections between written words and other language processes (Dehaene et al., 2010; Plaut and Behrmann, 2011). Our finding of bilateral fusiform recruitment for children who are still learning to read is in line with these predictions.

***The precentral gyrus***. Two of our results suggest that the left precentral gyrus is important for cursive letter perception after learning with self-generated action. First, our whole brain analysis revealed a trend toward greater recruitment of this area for perception of actively learned cursive letters compared to passively learned ones. Second, our ROI analysis showed that the left precentral gyrus was only recruited above baseline for the actively learned cursive letters, and not for the passively learned or unlearned cursive letters. The left precentral gyrus has been associated with letter perception in several studies and is thought to activate stored motor programs associated with letter production (Longcamp et al., 2005a, 2006b, 2011; James and Gauthier, 2006; Nakatsuka et al., 2012). This idea is supported by the James and Atwood (2009) results, which found that, in adults, motor experience (writing) with pseudoletters led to recruitment of this region, but when the pseudoletters were learned with only visual practice, the precentral gyrus was not recruited any more so than before learning took place. In this case, motor experience with the pseudoletters was essential for reactivation of the precentral gyrus during later perception. A similar result was shown by James and Engelhardt (2012) that only self-generated printing led to frontal motor system recruitment in preschool children during subsequent letter perception. Another study found that for lefthanders the right precentral gyrus was recruited during perception of printed letters, but not for unlearned pseudoletters, again suggesting that the motor experiences involved in production of letter are essential in order for the precentral gyrus to be recruited during letter perception (Longcamp et al., 2005a). This study is particularly telling because the motor activation occurred in the hemisphere contralateral to the hand used to write and only for previously written stimuli, thereby emphasizing the importance of writing experience for the reactivation of this region during perception. The results of the present research add to these findings by showing that passive observation of a letter unfolding over time does not recruit the precentral gyrus above baseline, but production does, thus providing strong evidence for the idea that the motor cortex is only recruited after self-generated action (See also James and Swain, 2011).

One difference seen between this work and previous work is that in a previous study of cursive letters, Exner's area, not the precentral gyrus, was found to be recruited (Nakamura et al., 2012). However, this discrepancy could be attributed to three methodological factors. The first is the use of children vs. adults. It is possible that this response in Exner's area develops with experience reading and writing in cursive script. Thus, the participants in this study would not show this activation. Alternatively, this discrepancy could be due to the use of letters in the present study instead of words as in previous work. This would then suggest that Exner's area is recruited more in response to planning to write whole words rather than individual letters, while the precentral gyrus is where motor plans for individual letters might be processed. Finally, this discrepancy could be driven by the way the stimuli were presented. In the previous study, words were presented with a trajectory that matched how the letters would be written. In the current work, the letters were presented statically. Thus, the activation of Exner's area would be a result of watching the letters unfold rather than seeing static letters appear. This is in line with the previous study in which Exner's area was found to be sensitive to the temporal trajectory of the word. Cursive primes were only able to produce a priming effect in this region when the trajectory of the word was presented in the correct direction (Nakamura et al., 2012). If this is indeed the case, it might be interesting in future studies to explore how children in the beginning stages of learning cursive respond to a similar priming paradigm.

#### Additional regions recruited after active learning

Further, when we directly compared active to passive learning of cursive letters, greater recruitment of the bilateral insula and claustrum was shown during the perception of actively learned letters than passively learned letters. The left insula has been previously identified as a letter selective region during perception of letters compared to perception of objects (Joseph et al., 2003) and has been associated with letter naming (Joseph et al., 2006). Recruitment of the left insula is typically thought to be reflective of phonological processing (Fujimaki et al., 1999; Borowsky et al., 2006; Joseph et al., 2006). Although no auditory stimuli were presented in this study, it is still possible that the recruitment of the insula seen here is reflective of phonological processing if the children were sub-articulating the letters. Outright articulation is thought to involve the left insula (Baldo et al., 2011), while the right insula has been linked to imagining articulation of phonemes (Kato et al., 2007). Given the recruitment of these regions in the present study, it is possible that following sub-articulation of the letters, children were better able to phonologically process letters that they learned by writing than those that they learned by observing an experimenter write. Further, because the right insula was revealed when comparing printed letters to passively and unlearned cursive letters, but not when comparing printed letters to actively learned cursive letters, this would suggest that writing practice has led to more similar neural representation between printed letters and those letters learned by writing.

Additionally, the insula and claustrum have been associated with multisensory integration (Hadjikhani and Roland, 1998; Naghavi et al., 2007) and postulated to act as a center for coordinating sensory and motor information, both within and across modalities (Crick and Koch, 2005). Interestingly, writing itself is a multimodal act that involves sensory input, (i.e., the visual feedback of the letter unfolding and the tactile sensations of holding a pencil or pen and touching paper) and motor execution. In fact, the multimodal aspects of writing reflect the key differences between the active and passive learning conditions in this study, so it would not be surprising if this difference manifests in multimodal integration regions of the brain. Although the task during the scanning session in the present study was not of multimodal nature, it is possible that just as letter perception has been found to activate the motor cortex (James and Gauthier, 2006; Longcamp et al., 2006b), other areas associated with writing may also be reactivated. As the claustrum has not been implicated in previous imaging studies of writing (James and Gauthier, 2006), future studies are warranted to address the specific role of the claustrum during writing.

#### Cursive letters and printed letters

Contrary to research findings in adults, the perception of cursive letters did not differentially recruit any brain regions relative to perceiving printed letters in our child participants. The results reported by Longcamp et al. (2011), revealed several regions of the adult brain that were recruited more for handwritten cursive letters than for printed ones, but no regions that were recruited more for printed letters. Several factors likely contribute to this difference in research findings. The first is that adults have had more extensive interactions with cursive letters. Whereas the children in this study had roughly 30 min of experience with the cursive letters, adults likely learned cursive formally in school and may even use cursive on a regular basis. In contrast, 7-year-old children interact with printed letters regularly. All children in this study received a score of at least 12 out of 15 on the printing evaluation and were reading printed words at or above an age-appropriate level, suggesting that these children were quite familiar with printed letters. However, they were only able to identify 56% of cursive letters prior to training. As a result, it is not surprising that perception of printed letters recruited regions more so than perception of cursive letters as children have much more experience with printed letters and specifically, more motor experience.

## Conclusions

Self-generated production of cursive letters during learning led to the recruitment of a sensori-motor network known to also be active during letter perception and reading, however, passive observation of a letter being formed did not. This finding adds to the growing literature suggesting that self-generated writing is important for setting up reading networks in the developing brain. Nonetheless, perceiving cursive letters did not affect brain activation any more than perceiving printed letters, suggesting that the motoric production of text stimuli is the crucial factor in creating this network, rather than the type of letter perceived or produced.

### Conflict of interest statement

The authors declare that the research was conducted in the absence of any commercial or financial relationships that could be construed as a potential conflict of interest.
